# Investigation of effect of filter on the stand‐up technique for total skin irradiation by Monte Carlo simulation

**DOI:** 10.1002/acm2.13119

**Published:** 2020-12-12

**Authors:** Wenchih Tseng, Ruiqi Li, Qiuwen Wu

**Affiliations:** ^1^ Department of Radiation Oncology Duke University Medical Center Durham NC USA

**Keywords:** electron dosimetry, Monte Carlo simulation, total skin electron irradiation

## Abstract

**Purpose:**

The aim of this study was to investigate dosimetric effects of scattering filter on the stand‐up technique for total skin irradiation (TSI) with a single electron field by Monte Carlo (MC) simulation.

**Methods:**

MC simulations were performed with BEAMnrc and DOSXYZnrc packages under EGSnrc environment. Scattering filter of a metal disc was mounted in the accessory slot. The filter materials (Cu, Fe, Au, Zn, Ag) were investigated, with thickness ranging from 0.05 to 0.55 mm, depending on material. The extended source to skin distance (SSD) ranging from 250 to 350 cm was studied. The following dosimetric quantities were evaluated: percent depth dose (PDD), profiles and output factor at depth of maximum, and composite dose distribution on a 30‐cm diameter cylindrical phantom. They were compared with the standard dual beam technique used at our clinic. The effects on different patient sizes were also studied.

**Results:**

No filter produced acceptable profile flatness (±10% within the central 160 cm) at 250 cm SSD. At 300 cm SSD, Au (0.1 mm), Ag (0.25 mm), Cu (0.5 mm) produced acceptable flatness while Zn (0.45 mm) required 325 cm SSD. For these four configurations, the d_max_ was 0.90–0.99 cm, similar to dual beam (0.97 cm); R_50_ was 1.85–1.91 cm, compared with dual beam of 2.06 cm; the output factor ranged from 0.025 to 0.029, lower than the dual beam (0.080). With the composite fields for four configurations, the d_max_ was 0.10 cm, compared with dual beam (0.16 cm). The surface dose was 97%, similar to dual beam (96%). B‐factor was 3.3–3.4, compared with dual beam of 3.1. The maximum X‐ray contamination was 3%, higher than dual beam (1%).

**Conclusions:**

The investigation suggests the TSI stand‐up technique can be implemented using a single electron beam if a customized filter is used. More dosimetric measurements are needed to validate the MC results and clinical implementation.

## 1 INTRODUCTION

Total skin Electron irradiation (TSEI or TSI) is an external beam therapy used to treat patients with malignant skin diseases such as mycosis fungoides or cutaneous T‐cell lymphoma. It is a special electron therapy technique that involves delivering a homogeneous radiation dose to the entire skin over the whole body within a limited depth (few millimeters),^1^ while sparing the radiation dose delivered to the organ at risks (OARs) beyond a few centimeters depth. To deliver a successful total skin electron therapy, the American Association of Physicists in Medicine Task Group No. 30 (AAPM TG‐30)^2^ recommends that the field size of the composite electron field must be approximately 200 cm in height by 80 cm in width at the treatment plane to cover large patients, the dose uniformity over the central 160 × 60 cm^2^ region should be within ±8% in vertical and ±4% in horizontal directions, and X‐ray contamination (1%) of the electron fields is desired.^2^, ^3^

Various TSI techniques have been developed and described in AAPM TG‐30.^2^ At our clinic, the standard procedure for TSI treatment is the Stanford six dual‐field method.^2^ To provide a uniform dose distribution on the patient, dual electron fields with ±19 degree angled from horizontal are directed at patient standing on the TSI platform at an extended source to skin distance (SSD) of 300 cm. Patient rotates along the craniocaudal axis in six directions: anterior to posterior (AP), right anterior oblique (RAO), right posterior oblique (RPO), posterior to anterior (PA), left posterior oblique (LPO), and left anterior oblique (LAO) with interval of 60 degree to get full dose coverage to entire skin over the whole body. The other procedure for TSI treatment implemented at our clinic is a TSI lay‐down/recumbent technique with a customized scattering filter based on Mayo Clinic.^4^ The lay‐down technique was first developed by Wu et al.^5^ and further modified by Deufel and Antolak^4^ with mounting an extra scattering filter at the exit window of the Linac. It is an alternative for frail patients who are too weak to stand in a certain position for a long time of setup and treatment. In the lay‐down technique, the patient's umbilicus is positioned under the Linac head for the vertex fields (AP/PA) and the oblique fields (RAO/LAO/RPO/LPO) with gantry angle of 60 degree are delivered with the patient positioned in a head to foot direction parallel to the Linac gantry rotation axis.

One key requirement in the lay‐down technique is the use of a customized scattering filter to broaden the electron field for compensating the reduced SSD for the vertex fields. Few research groups have implemented different designs of scattering filter for TSI techniques: Pham et al.^3^ designed an aluminum/polystyrene electron scattering filter for a single field rotational total skin irradiation to redistribute the electron beam; Podgorsak et al.^7^ constructed an electron beam degrader made of lead/aluminum; El‐Khatib et al.^8^ designed a beam scattering filter from Lucite; Reynard et al.^9^ built a custom flattening filter constructed of aluminum, lead, and polymethyl methacrylate used in rotational total skin electron irradiation.

According to the previous studies, it could be more efficient to produce an equivalent beam uniformity by a scattering filter with a complicated design and variations of material and thickness. However, it could be difficult to construct a complicated design of scattering filter made out of several layers of material with different dimensions accurately. In this study, we investigated the dosimetric effect of a scattering filter with a simple circular design on a modified stand‐up technique where only a single electron field is used at each direction. This technique with a scattering filter mounted could eliminate the use of dual beam in the standard stand‐up technique, improve the uniformity of a single treatment field, and potentially improve the efficiency of treatment setup.

Current commercial treatment planning systems are unable to perform the TSI treatment planning. Furthermore, several nonstandard measurements and dosimetric procedures involved in the both commissioning of the TSI techniques and patient treatment are very time‐consuming and limited to a point dose only, which have made it difficult to develop and perform on a routine basis. Therefore, Monte Carlo (MC) simulation which has been found useful for reference and dosimetric calculations is best suited for the validation of the commissioning results and providing more information on dose distribution. Recently, we have successfully built a MC model under EGSnrc environment and used to validate both TSI stand‐up and lay‐down techniques.^6^ Since MC simulation can provide more information such as a full dose distribution on a phantom other than a point dose measurement, it can be a useful guidance for further optimization of the TSI treatment technique. In this study, the dosimetric effect of the scattering filter on the modified stand‐up technique was investigated with our previously validated MC model.

## 2 MATERIALS AND METHODS

In this section, the design of the scattering filter and the description of the modified TSI stand‐up are first presented. The MC model implemented under EGSnrc environment to investigate the dosimetric effect of the scattering filter is then described in detail. Furthermore, dosimetric metrics used to analyze and compare the standard and modified TSI techniques are discussed. The experimental measurement for the modified technique is also described in the end of this section.

### 2.A Description of the modified TSI technique

#### 2.A.1 Scattering filter design

**FIG. 1:**
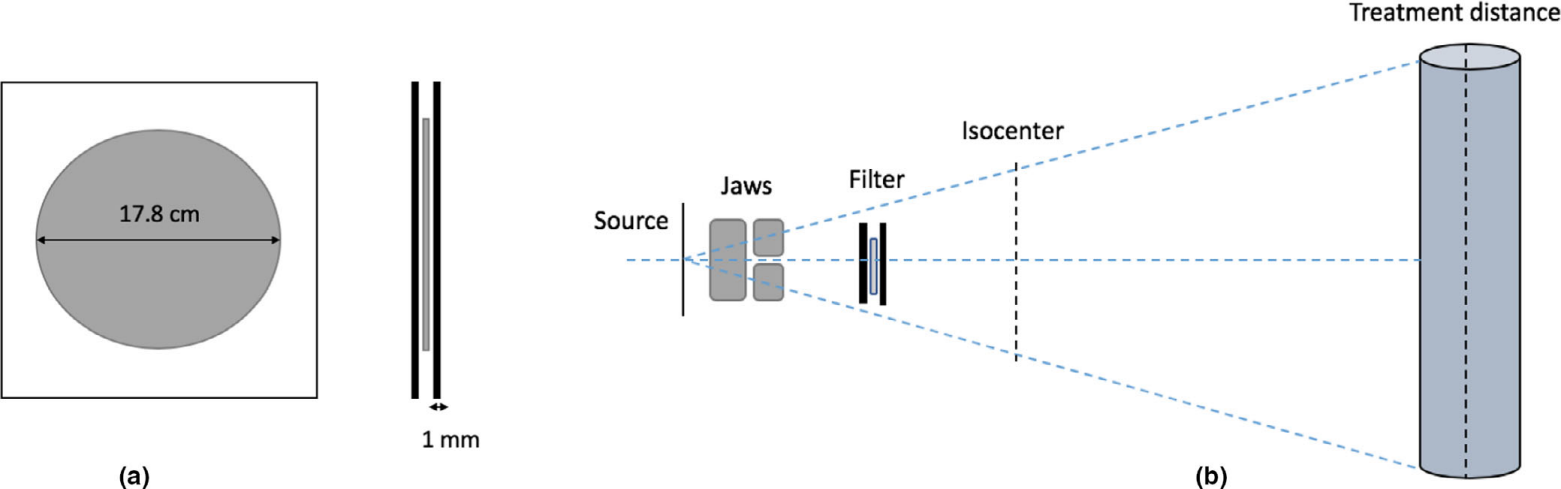
(a) Top and side view of scattering filter: constructed by two 1 mm layers of polycarbonate and a 17.8 cm diameter circular metal disc. (b) EGSnrc MC model: beam source, X/Y Jaws, scattering filter, and a phantom at treatment distance were included in MC simulation.

The customized scattering filter used in this study was based on the design of the scattering filter originally from Mayo Clinic,^4^ constructed by a 0.25‐mm thickness copper disc placed between two 1 mm polycarbonate layers and implemented for a TSI lay‐down technique at our clinic. The detailed geometry of the scattering filter is shown in Fig. 1(a).

To find out the optimal configuration of scattering filter, various filter material, filter thickness, and setup SSD were studied (Table 1) The metals, iron (Fe), gold (Au), silver (Ag), zinc (Zn), and copper (Cu) were chosen since they are high atomic number material which are readily available on the market and physically and chemically stable at room temperature. The filter thickness from 0.05 to 0.55 mm and the setup SSD from 250 to 350 cm were studied. For each material with different thickness, the profile flatness at each extended SSD was examined to evaluate if it can achieve the requirement recommended by AAPM TG‐30.^2^

**TABLE 1:** Configurations of filter material, thickness, and setup SSD were studied.

Material	Fe	Au	Ag	Zn	Cu
SSD (cm)	250–350				
Thickness (mm)	0.05–0.55				

#### 2.A.2 Modified TSI stand‐up technique

The customized scattering filter was mounted onto the accessory slot of the Linac (57.5 cm from the beam target). Instead of six pairs of electron fields (upper and lower fields at each direction) that were used in the standard technique, six single electron fields were directed to phantom standing at an extended SSD in the modified stand‐up technique. Phantom was rotated along the cranial‐caudal axis on a TSI platform in six positions with 60° interval to get full dose coverage to skin. For each direction, a single electron field with jaws opening (X/Y Jaws) at 30 × 40 cm^2^, collimator rotation at 90°, and fully retracted multileaf collimator (MLC) were used for the modified stand‐up technique.

### 2.B Monte Carlo simulation

In our previous study,^6^ we built a MC model that has been successfully used to validate both TSI stand‐up and lay‐down techniques. The same MC model was implemented in this study to investigate the dosimetry of the scattering filter on the modified stand‐up technique.

#### 2.B.1 Phase space files

The Linac vendor (Varian Medical System, Inc., Palo Alto, CA) does not release the geometric and material information upstream of the secondary collimator (X and Y Jaws) for the TrueBeam Linac because of proprietary confidentiality.^10^ Instead, phase‐space files compatible with the International Atomic Energy Agency (IAEA) format for clinical electron energies above the jaws are provided by the vendor for research use, available on www.MyVarian.com. In this study, the phase‐space file of 6 MeV electron beam (version 2) for TrueBeam Linac, which was originally recorded at a plane above the moveable jaw with 26.7 cm away from the beam target and 73.3 cm from the isocenter using GEANT4 (version 10.0. patch1) based code, was used as the main beam source in EGSnrc MC simulations.^11^

#### 2.B.2 EGSnrc: Monte Carlo model and parameters

Monte Carlo simulations in this study were performed with BEAMnrc^12^ and DOSXYZnrc^13^ packages under EGSnrc environment which has had a significant lead in the radiation therapy field. The BEAMnrc allows for the simulation of any components in the Linac and particles transport. The DOSXYZnrc is to calculate the three‐dimensional (3D)‐deposited dose distribution within an arbitrary voxelized phantom. The EGSnrc code has been validated in our previous study on TSI and seemed best suited for this study of techniques optimization. Photon global cutoff energy (PCUT) and electron global cutoff energy (ECUT) were set to 0.01 and 0.521 MeV, respectively. ESAVE GLOBAL was 1 MeV. These parameters and algorithms of particles transport recommended by the previous publication^14^ were chosen to balance between the accuracy and efficiency of the MC simulations.

The Varian TrueBeam STx Linac model was accurately built based on the geometry of Linac head provided by the manufacturer using predefined component modules (CMs) in BEAMnrc code. The schematic diagram of the MC model based on the clinical Linac is shown in Fig. 1(b). In our study, the Linac geometry for the 6 MeV electron beam was composed of secondary collimators (X and Y Jaws) and the scattering filter which were modeled by JAWs and SLAB/FLATFILT CMs, respectively. A CM of SLAB was specified for the position of phase‐space file which served as a main beam source. A scoring plane, where any particle traveling through it will be recorded and stored in an output phase‐space file, can be defined and placed under any pre‐built CMs.

To simulate the modified stand‐up technique, scoring planes were defined in BEAMnrc and placed at 58 cm (Linac exit window), 250, 300, 325, and 350 cm from the beam target. phase‐space files generated at the Linac exit window was used to analyze the beam characteristic using BEAMDP code (BEAM Data Processor).^15^ The others generated at extended SSDs (from 250 to 350 cm) were served as inputs in DOSXYZnrc to calculate 3D dose distributions in a 200 × 100 × 10 cm^3^ flat solid water phantom with a voxel size of 2.0 × 2.0 × 0.1 cm^3^. The output .3ddose files were read by an in‐house built MATLAB code to extract 3D dose distributions on the phantom. Profile flatness for each material and thickness can be computed from the 3D dose distribution to determine the optimal filter configurations.

To visualize the dosimetric effects of the composite field, a 30‐cm diameter cylindrical solid water phantom with a length of 180 cm and a voxel size of 0.1 × 1.0 × 0.1 cm^3^ was generated by a Python code and imported to DOSXYZnrc for dose calculation. A single electron field in the modified stand‐up technique was simulated in BEAMnrc. Three‐dimensional dose matrixes extracted from the output .3ddose file of DOSXYZnrc were then rotated (to simulate six directions) and combined together to get a composite 3D dose distribution. Furthermore, cylindrical phantoms with different diameters (20, 25, 35, 40 cm) were simulated to evaluate the dosimetric effect on patient sizes.

### 2.C Comparison metrics

To evaluate the effect of the scattering filter, dosimetric metrics such as beam profiles at depth of maximum dose, d_max_, percentage depth dose (PDD), and output factor were computed and compared with the simulated data for the standard TSI stand‐up technique that has been used at our clinic. The study of these dosimetric metrics were conducted by EGSnrc MC simulations, with some actual measurements in phantom to validate MC results (Section 2.D).

#### 2.C.1 Phase‐space file analysis

Mean energy distribution and angular distribution of phase‐space file generated at the Linac exit window (57.5 cm from beam target) were analyzed to evaluate the effect of an existence of the scattering filter on beam characteristics using BEAMDP.

#### 2.C.2 Longitudinal/lateral profiles and Planar dose distribution

3D dose matrices extracted from .3ddose file were postprocessed by an in‐house built MATLAB code. Simulated longitudinal and lateral profiles at 1 cm depth of fields with scattering filter at treatment distance were calculated by averaging the voxels located at two neighboring depth and then normalized to the dose value at central axis. Planar dose distribution at 1 cm depth can also be computed from the 3D dose matrix by normalizing each voxel to the dose value at central axis. The flatness of these profiles was evaluated based on the requirement recommended by AAPM TG‐30.

#### 2.C.3 Percentage depth dose

Percentage depth doses were calculated by averaging the neighboring four voxels at the same depth and then normalized to the maximum dose. Surface dose, depth of maximum dose (d_max_), depth of 80% maximum dose (R_80_), depth of 50% maximum dose (R_50_), X‐ray contamination at 10 cm depth, and body factor were calculated for the comparison between the standard and modified techniques.

#### 2.C.4 Output factor

The output factor for the technique can help compare the treatment efficiency and determine the monitor units (MUs) needed to deliver a prescribed dose. The relative output factor^6^ can be determined by scaling the ratio of charge measured at treatment distance with treatment setup to the charge measured at the reference calibration condition with field size of 36 × 36 cm^2^, depth of 1.5 cm, and removal of an applicator. The output factor at surface on a flat solid water phantom was calculated and then converted to the absolute dose used for MU calculation. The difference of the output factors between the standard and modified techniques was computed.

#### 2.C.5 Body factor and X‐ray contamination

The X‐ray contamination of the composite fields is defined as the ratio of the dose at the central voxel of the central slice to the dose at the surface of the cylindrical phantom. Body factor (B‐factor), defined as the ratio of output of the surface voxel at central axis from six directions to a single direction. The difference of B‐factor and X‐ray contamination between the dual open field used in the standard technique and a single filtered field used in the modified technique was calculated and compared.

#### 2.C.6 Investigation of the dosimetric effect on patient sizes

To evaluate the effect of patient sizes on dosimetric result for the modified technique, cylindrical phantoms with diameters of 20, 25, 30, 35, and 40 cm were simulated to calculate 3D dose distributions. In this study, a general patient size was assumed of 30 cm and the position (SAD) of the origin of the patient was fixed. That is, SSD increases when patient size decreases. The dosimetric metrics which may potentially vary with the patient size such as X‐ray contamination and B‐factor were mainly compared and discussed.

### 2.D Actual measurement verification

Most of the results in this study were produced by EGSnrc MC simulations. After several MC simulations, a few configurations of filter design satisfied the recommendations of AAPM TG‐30 for beam uniformity of TSI. We chose one of them to do an actual measurement (Cu 0.5 mm filter) in phantom to validate the MC result. The measurement was performed on a Varian TrueBeam STx Linac in the 6 MeV HDTSe mode with a dose rate of 2500 MU/min. A research scattering filter was constructed by a circular copper disc with a thickness of 0.5 mm and a diameter of 17.8 cm, and a thickness of 3 mm hexagon shape polycarbonate layer. In the measurement, the scattering filter was mounted onto the accessory slot of Linac. Field size of 30 × 40 cm^2^ (X and Y Jaws), collimator rotation of 90°, and SSD of 100 and 300 cm were used without an electron applicator. Longitudinal and lateral profiles of 1 cm depth, central axis PDDs, and output factor at surface were measured by a plane‐parallel ion chamber on a flat solid water phantom.

## 3 RESULTS

### 3.A Linac head modeling result

**FIG. 2:**
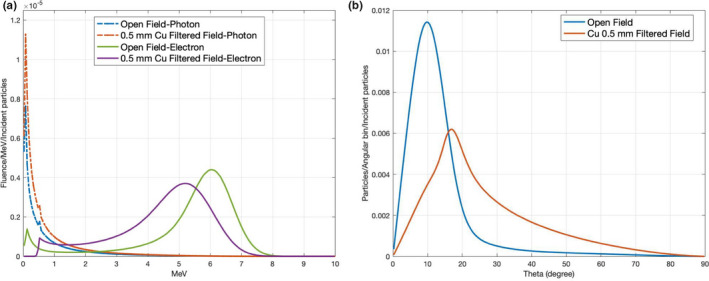
The effect of 0.5‐mm copper filter on beam characteristic. (a) Energy spectrum of photon (dashed) and electron (solid) for the beam with/without copper filter at the exit window of the Linac head (SSD = 57.5 cm). (b) Angular distribution of electrons for the beam with/without copper filter at SSD = 57.5 cm.

Figure 2(a) shows the energy spectrum of electrons and photons, and Fig. 2(b) shows the angular distribution of the incident electrons recorded at the Linac exit window for both open and filtered field in MC simulations, respectively. Compared to the open field, the mean electron energy decreased and photon contamination increased for the filtered field, the electrons also spread more with the filter. The statistical uncertainty of the MC simulation was around 0.8% at d_max_, 1.5% at fall‐off region, and 5–15% in the low dose bremsstrahlung region.

### 3.B Results in a flat water phantom

**FIG. 3:**
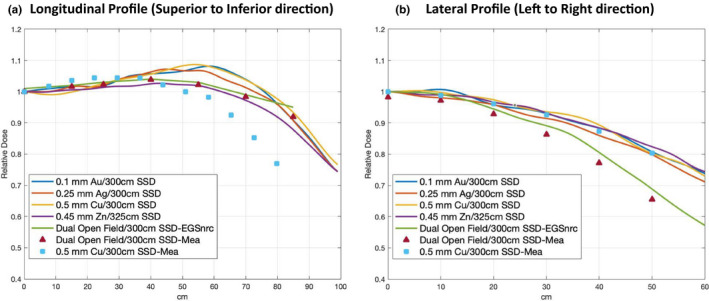
(a) Longitudinal (from patient's superior to inferior) profiles comparison of 1 cm depth at extended SSD for four filter configurations (solid lines) and dual open field (marker). Doses were normalized to the value of central axis. (b) Lateral (from patient's left to right) profiles comparison of 1 cm depth at extended SSD for four filter configurations (solid lines) and dual open field (marker). Doses were normalized to the value of central axis.

**FIG. 4:**
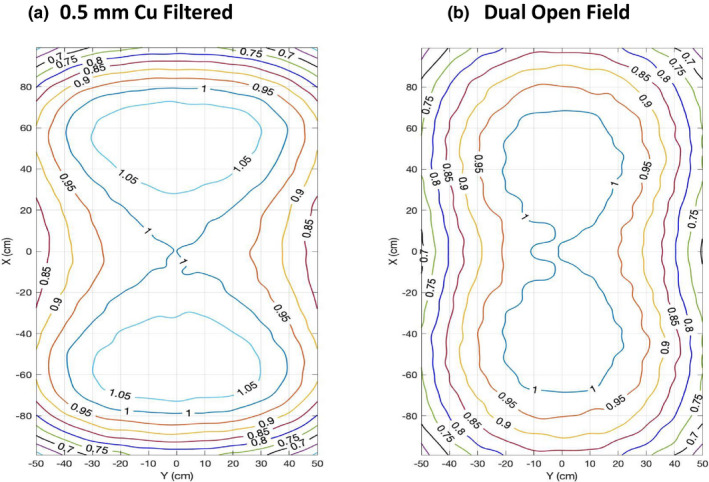
Planar dose distribution at 1 cm depth in a flat solid water phantom at extended SSD for (a) a single 0.5 mm Cu filtered field and (b) dual open fields. Doses were normalized to the value at central axis. The four configurations demonstrated similar planar dose distributions.

The simulated longitudinal and lateral profiles on a flat solid water phantom are shown in Figs. 3(a) and 3(b). Profiles were calculated by averaging the voxels located at two neighboring depth and then normalized to the dose value at central axis. Four configurations (Table 2), 0.1 mm Au/300 cm SSD, 0.25 Ag mm/300 cm SSD, 0.5 Cu mm/300 cm SSD, and 0.45 mm Zn/325 cm SSD, can produce a profile flatness recommended by AAPM TG‐30. There was a huge discrepancy between the measurement and simulation of the 0.5 mm Cu filter in the longitudinal direction. This will be discussed in the Section 4.B. Compared to the dual open field, these four filter configurations with a single electron field can produce a better dose uniformity in lateral direction, which can be clearly seen in the planar dose distribution shown in Fig. 4.

**TABLE 2:** The four configurations satisfied the criteria of beam uniformity recommended by TG −30.

Material	Au	Ag	Zn	Cu
SSD (cm)	300	300	325	300
Thickness (mm)	0.10	0.25	0.45	0.50

Planar dose distributions of the fields at 1 cm depth on a flat solid water phantom are shown in Fig. 4, doses in each voxel were normalized to the average dose at central axis. Within ±10% difference from the prescription dose at central axis, about a central 170 × 100 cm^2^ field and 180 × 80 cm^2^ field can be produced by the single filtered field with 0.5 mm Cu and the dual open field, respectively. They both passed the requirement of a central 160 × 60 cm^2^ field recommended by AAPM TG‐30. Please note that the four configurations produced a similar planar dose distribution, but only the one with 0.5 mm Cu filter is shown and compared with dual open field here.

### 3.C Results in a cylindrical phantom

**FIG. 5:**
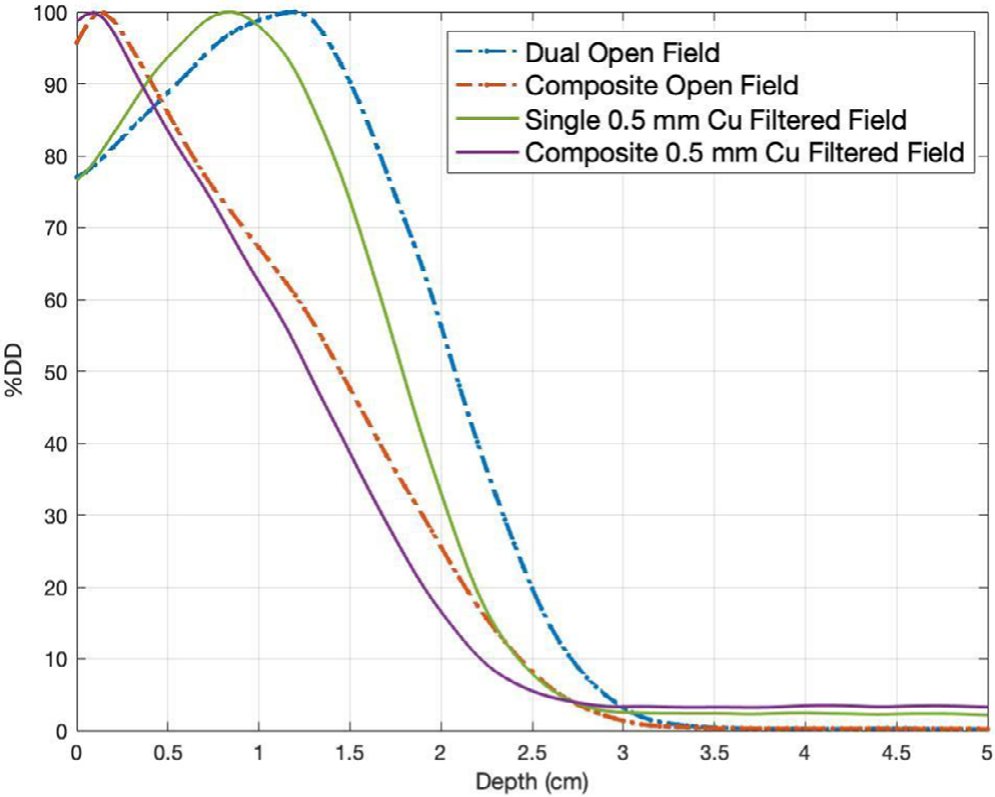
Percentage depth dose curves (from MC simulations) at the central slice of cylindrical phantom for single and six directions.

In the cylindrical phantom, simulated PDDs of individual field and composite fields are shown in Fig. 5. The d_max_ of both composite PDDs shifted toward the surface of the cylindrical phantom from the single direction. The filtered fields used in the modified stand‐up technique can produce a relatively higher surface dose, but also result in a higher X‐ray contamination, compared to the standard stand‐up technique. The detailed comparison of surface dose, d_max_, R_80_, R_50_, and maximum X‐ray contamination between the standard and modified stand‐up techniques are listed in Table 3.

**TABLE 3:** Dosimetric quantities of single and composite central axis PDDs at treatment distance for the dual open field and a single Cu filtered field on a 30‐cm diameter cylindrical phantom.

Percentage depth dose	Surface dose (cm)	d_max_ (cm)	R_50_ (cm)	R_80_ (cm)	Maximum x‐ray contamination (% of D_max_)
Single dual open field	77.0	1.15	2.07	1.67	0.55
Single Cu filtered field	76.6	0.90	1.85	1.41	1.60
Composite dual open field	95.8	0.15	1.45	0.64	1.29
Composite Cu filtered field	97.3	0.10	1.27	0.58	3.35

The relative output factor at 1.5 cm depth at 100 cm SSD for the field with 0.5 mm Cu scattering filter was 0.221 cGy/MU in MC simulation, compared to 0.215 cGy/MU in measurement. At 300 cm SSD, the relative output factor at surface for the filtered field with 0.5 mm Cu was 0.021 cGy/MU (MC) and 0.020 cGy/MU (Measurement). The output factor at 1 cm depth at treatment distance for the four configurations ranged from 0.025 to 0.029 cGy/MU. For the composite field of the four configurations, the d_max_ was 0.10 cm, compared to 0.16 cm in the standard dual‐field technique. The maximum surface dose was 96–97% in the modified technique, similar to dual open fields (96%) in the standard technique. B‐factor was 3.3–3.4, compared with dual open field (3.1). The maximum X‐ray contamination in standard and modified technique were 1% and 3%, respectively.

**FIG. 6:**
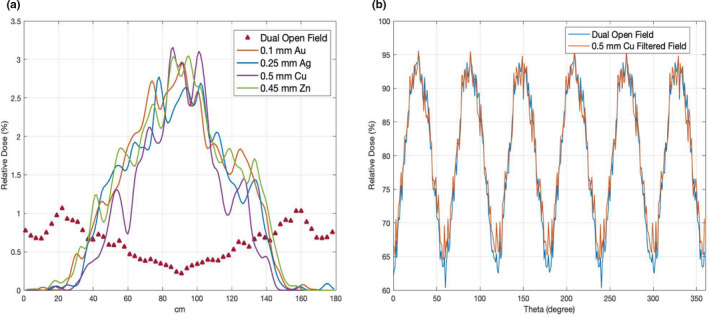
(a) X‐ray contamination (%) along superior to inferior direction of cylindrical phantom for composite dual open field (marker) and composite filtered field (solid lines). (b) Surface dose (%) of the central slice on a 30‐cm diameter cylindrical phantom for the standard and modified TSI stand‐up techniques.

Figure 6(a) shows the X‐ray contamination along the phantom's superior to inferior direction, and Fig. 6(b) demonstrates the surface dose of central slice of the 30‐cm diameter cylindrical phantom for the composite dual open field (Standard technique) and Cu filtered field (Modified technique). The maximum X‐ray contamination for the composite single filtered field (3%) was located at the central region of the phantom while the maximum X‐ray contamination for the composite dual open field (1%) was located at the edges of the phantom. Both composite filtered field and open field can produce a similar surface dose of central slice on the cylindrical phantom.

**FIG. 7:**
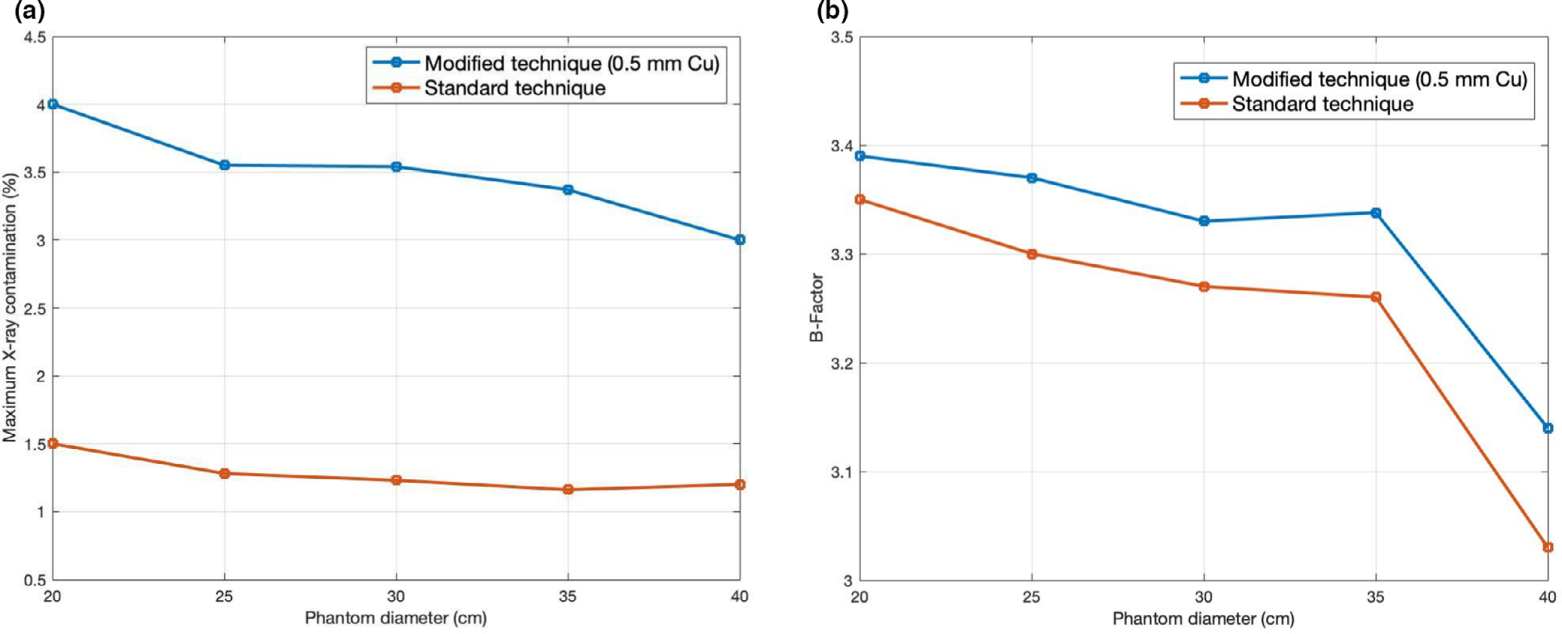
(a) Maximum X‐ray contamination (%) for both modified and standard techniques with different phantom sizes (20, 25, 30, 35, 40 cm). (b) B‐Factor for both modified and standard techniques with different phantom sizes (20, 25, 30, 35, 40 cm).

Maximum X‐ray contamination and B‐factor on different phantom sizes (20, 25, 30, 35, 40 cm) for both standard and modified techniques are shown in Figs. 7(a) and 7(b), respectively. In general, the B‐factor of different phantom sizes for both techniques had a similar trend. That is, B‐factor decreased when phantom size increased. Likewise, the maximum X‐ray contamination decreased when phantom size increased for both techniques.

## 4 DISCUSSION

### 4.A Comparison between the standard and modified TSI stand‐up techniques

Four configurations (Table 2): 0.1 mm Au (300 cm SSD), 0.25 mm Ag (300 cm SSD), 0.5 mm Cu (300 cm SSD), and 0.45 mm Zn (325 cm SSD) satisfied the passing criteria (±10% profile flatness over a central 160 × 80 cm^2^ region) at extended SSDs. They can produce a similar planar dose distribution of an effective 170 × 100 cm^2^ field size while standard stand‐up technique can provide an effective 180 × 80 cm^2^ field size. The modified technique can achieve a better equivalent square field size (126 × 126 cm^2^), compared to 110 × 110 cm^2^ in the standard technique.

While the d_max_ of composite PDDs for both techniques shifted toward surface from a single direction, a higher surface dose can be produced with the scattering filter mounted, compared to the standard technique. This shows that the modified technique may be targeted for shallower lesions while the standard technique is for relatively deeper lesions.

In the modified technique, there was a hump region at the off‐axis distance of 60 cm in the longitudinal direction shown in Fig. 3(a). This was produced by the scatter from the edge of the scattering filter. The longitudinal dose profiles for the modified technique still can produce an acceptable flatness recommended by TG‐30, even though the dose dropped relatively faster at the off‐axis distance of 80 cm than the standard technique. However, this may potentially make head and foot underdosed. Therefore, extra boosted fields which are commonly required in the standard technique are also needed for the underdosed areas in the modified technique. Since the field coverage in the lateral direction is wider in the modified technique than the standard technique, the modified technique could be targeted for stocky patients.

It may be surprising that the four configurations using a single field with filters produced a better lateral profile flatness than the standard dual open field technique, as shown in Fig. 3(b). Thanks to MC's capability to provide the volumetric dose distribution, this becomes easy to understand from the planar dose distribution shown in Fig. 4. Because the lateral profiles were measured on the central slices, which is contributed equally by the edges of the two overlapping fields, rather than the center of each field, this caused the nonoptimum lateral profile flatness but optimal flatness in the longitudinal axis. This shows a benefit of using MC simulation rather than the traditional point‐based dose measurement and demonstrates the advantages of using MC as a tool to design and commission such special treatment techniques.

The scattering filter made out of material with high atomic number in the modified technique can degrade the electron energy and increase the angular distribution of the electrons which allows to produce a large electron field. That is, it can eliminate the use of overlapping fields in the standard technique, with comparable dosimetric characteristics. Furthermore, the reduction of the field junction from dual fields to a single field for each direction in the modified technique would avoid hot spots or overdosed regions on the patient.

However, the scattering filter can also introduce secondary photons which will produce a higher photon contamination in the patient. The overall effect depends on the filter and the beam arrangement. The modified technique produced a maximum X‐ray contamination of 3% due to the effect of the scattering filter on beam characteristic (shown in Fig. 2), slightly higher than the standard technique (1%) with dual open fields, which was intentionally angled away to minimize the X‐ray contamination on the central axis. In our lay‐down technique with 0.25 mm Cu filter, the maximum X‐ray contamination was about 2%. Because only single field per direction was used in the modified technique, the X‐ray contamination was highest at the central slice, but it quickly decreased with the off‐axis distance. For example, at the off‐axis distance of 10 cm, the X‐ray contamination dropped to 2%, as shown in Fig. 6. Nevertheless, a further evaluation of X‐ray contamination on the modified technique should be investigated to find out if a higher X‐ray contamination would result in any extra side effects on patients.

As mentioned in the result section, the modified technique had a lower output factor (0.025–0.029 cGy/MU) at extended SSDs, compared with 0.08 cGy/MU in the standard technique. To deliver the same amount of prescription dose to the patient skin, the total MU needed in the modified technique is about 1.2‐ to 1.5‐fold, compared to the total MU needed in the standard technique. That is, the efficiency of the treatment might not be improved with the scattering filter mounted, even though there is no field junction needed in the modified technique. However, given the fact that the dose rate for the HDTSE is at 2500 MU/min, the overall treatment time increase is not significant. In comparison, the standard lay‐down technique needs about two times of MU to deliver the same amount of prescription dose, compared with the standard Stanford technique.

As a conclusion, with the similar dosimetric results simulated by EGSnrc, the modified technique could be an alternative in the TSI stand‐up treatment, especially targeted for those patients whose lesions are located at relatively shallower depth.

### 4.B Dose discrepancy on the beam profile between the measurement and MC result

As shown in Fig. 3(a), there was a fairly large difference in the longitudinal dose profile between the measurement and MC results for the 0.5 mm Cu filtered field, up to 10% at 60 cm away from central axis. In our previous study, the MC results for the lay‐down technique which also implemented a scattering filter in the MC model in general agreed well with the measurement. Therefore, the dose discrepancy on the profile might be caused by the research scattering filter (0.5 mm Cu) and the mounting plates used in the measurement. The 0.5 mm Cu disc from Amazon.com might not be suitable for this study, even though it was rated at 99.9 % purity. That is, its purity and exact geometry might not be as good as the scattering filter clinically used for the lay‐down technique. Therefore, more accurate constructions in terms of filter thickness, mounting plates, and pure material from a professional manufacturer and rigorous measurements are needed if the modified technique is clinically implemented.

The uncertainty for the MC simulation included room scatter effects^16^ from walls, floor, ceiling, and TSI platform in the treatment room since the MC model cannot account for every detailed geometry. Furthermore, the measurements done at the treatment plane can be hard to set up accurately. That is, any variations of SSD, angular position, and off‐axis distance at an extended SSD could bring uncertainty to the measurement. Therefore, the uncertainties from both MC simulation and measurement mentioned above could potentially lead to the discrepancy shown on the dose profiles.

## 5 CONCLUSIONS

The results of our Monte Carlo calculations found that the TSI stand‐up technique can be implemented using a single electron field if a customized filter is used, with producing comparable dosimetric results as the standard technique that has been used at our clinic. Monte Carlo simulation is valuable in performing this type of investigation, it reduces the need of measurement considerably. In addition to those measurable quantities, the Monte Carlo simulation can provide further investigations such as a full dose distribution of the patient phantom, and the ability to investigate and optimize techniques such as different filter designs, SSD, and field size variations.

## CONFLICT OF INTEREST

There is no conflict of interest for all authors.
